# Bladder Cancer Cells Interaction with Lectin-Coated Surfaces under Static and Flow Conditions

**DOI:** 10.3390/ijms24098213

**Published:** 2023-05-04

**Authors:** Renata Szydlak, Ingrid H. Øvreeide, Marcin Luty, Tomasz Zieliński, Victorien E. Prot, Joanna Zemła, Bjørn T. Stokke, Małgorzata Lekka

**Affiliations:** 1 Department of Biophysical Microstructures, Institute of Nuclear Physics, Polish Academy of Sciences, PL-31342 Kraków, Poland; renata.szydlak@ifj.edu.pl (R.S.); marcin.luty@ifj.edu.pl (M.L.); tomasz.zielinski@ifj.edu.pl (T.Z.); joanna.zemla@ifj.edu.pl (J.Z.); 2 Biophysics and Medical Technology, Department of Physics, The Norwegian University of Science and Technology (NTNU), NO-7491 Trondheim, Norway; ingrid.h.ovreeide@ntnu.no; 3 Biomechanics, Department of Structural Engineering, The Norwegian University of Science and Technology (NTNU), NO-7491 Trondheim, Norway; victorien.prot@ntnu.no

**Keywords:** urothelial and bladder cancers, adhesion, lectin–glycan interaction, single cell force spectroscopy (SCFS), microfluidics, cell sorting

## Abstract

Aberrant expression of glycans, i.e., oligosaccharide moiety covalently attached to proteins or lipids, is characteristic of various cancers, including urothelial ones. The binding of lectins to glycans is classified as molecular recognition, which makes lectins a strong tool for understanding their role in developing diseases. Here, we present a quantitative approach to tracing glycan–lectin interactions in cells, from the initial to the steady phase of adhesion. The cell adhesion was measured between urothelial cell lines (non-malignant HCV29 and carcinoma HT1376 and T24 cells) and lectin-coated surfaces. Depending on the timescale, single-cell force spectroscopy, and adhesion assays conducted in static and flow conditions were applied. The obtained results reveal that the adhesion of urothelial cells to two specific lectins, i.e., phytohemagglutinin-L and wheat germ agglutinin, was specific and selective. Thus, these lectins can be applied to selectively capture, identify, and differentiate between cancer types in a label-free manner. These results open up the possibility of designing lectin-based biosensors for diagnostic or prognostic purposes and developing strategies for drug delivery that could target cancer-associated glycans.

## 1. Introduction

Genetic mutations and molecular alterations on the cell surface are one of the various fundamental features of cancer cells [1]. Among others, they manifest a change in cell–cell and cell–extracellular matrix (ECM) interactions [2,3,4] that are associated with alterations in the expression of adhesion molecules present in the plasma membrane, such as cadherins, integrins, selectins, or immunoglobulin-like superfamily [2,3]. These molecules frequently bear carbohydrate moieties (i.e., glycans) that actively regulate cell–cell and cell–ECM interactions [4,5]. Alterations in the type and expression of glycans observed during cancer development lead to abnormal glycosylation of proteins [6,7,8], which occurs very early during carcinogenesis, before cell differentiation or proliferation changes can be detected [9]. For many types of cancers, changes in glycosylation patterns contribute to the transformation to the malignant phenotype by cells, the ability to metastasize, changes in adhesion properties, and the rate of proliferation [10,11,12]. Several research groups have already studied the glycosylation pattern of bladder cancer cells [10,13,14,15,16,17]. For example, in bladder cancer cells, cadherin N-glycosylation has been associated with the invasive potential of the cells [10]. Guo et al. demonstrated that glycans change globally during the epithelial-mesenchymal transition (EMT) induced by transforming growth factor β (TGF-β) in non-malignant HCV29 cells [14]. In particular, bi-antennary and tetra-antennary N-glycans structure truncation has been observed. Another example demonstrated a decrease in the expression level of glycans that could be recognized by the lectins DBA (*Dolichos Biflorus Agglutinin*) and LCA (*Lens Culinaris Agglutinin*) [14]. DBA binds mainly to the N-acetyl-α-D-galactosamine (GalNAc) complex, which is associated with developing neoplasms [15]. Some studies have shown that DBA affinity to cell glycans is much higher for normal bladder cells than for cancer cells [18]. The lectin WGA (wheat germ agglutinin from *Triticum Vulgaris*) interacts with the sialylated glycoproteins but may bind to GlcNAc [13]. The presence of GlcNAc, as well as hypersialization, often accompanies the increase in tumor invasiveness [16]. The lectin PHA-L (phytohemagglutinin-L from *Phaseolus Vulgaris*) interacts with GlcNAc, often present on the surface of T24 and HT1376 cells [10,19]. These and many other examples [20] illustrate that recognition of cells based on lectin–glycan interaction is sufficiently specific to identify cancer-related changes. In some cases, glycosylation patterns undergo modification during carcinogenesis and can be used as a biomarker of malignancy.

The detection of cell surface glycans can be realized using various biological techniques, such as Western blotting [10], microarray screening [21], enzyme-linked lectin sorbent assays (ELISA) [22], fluorescence microscopy [23], flow cytometry [24], and high-performance liquid chromatography (HPLC) [25]. The most common approach uses lectins (i.e., plant glycoproteins) to recognize a specific glycan structure attached to various surface receptors [26]. The interest in exploiting glycans’ potential in cancer research has increased in recent years [27,28], focusing, for instance, on the direct effect of lectins as drugs that induce cell deaths [29,30,31]. Several attempts have used lectins as the main molecules in biosensor development for cancer cell recognition at the single-cell level [23,32,33]. However, the exact knowledge of how adhesive properties change during static and flow conditions is necessary to fully exploit the lectin–glycan interactions as biomarkers of various cancer types, including bladder cancer.

The adhesive properties of cells change dynamically depending on various stimuli, growth conditions, and functional or pathological stages of cells [34,35]. In parallel to commonly studied integrins’ or cadherins’ involvement in cell adhesion, glycans attract attention due to aberrant glycosylation of proteins and lipids observed in various cancers [36]. Cancer-specific changes can serve as novel targets for various applications, such as targeted tumor imaging [37]. These reports motivate the idea of using lectin-coated surfaces in microfluidic devices to capture cells similar to those demonstrated for cancerous B and T lymphocytes flowing through a post-device, resulting in the lectin-type-dependent attachment of cells [38]. In its assumption, capturing cancer cells with altered deformability and adhesiveness will allow the sorting of cancer cells by passing mechanically distinct cells to a chamber with a lectin-coated surface to which cells with specific adhesive properties will attach. Such an approach could potentially extend the already existing microfluidic devices tested, such as real-time deformability cytometry (RT-DC, [39,40]) and others [41,42,43], with such sorting capability. However, gaining more profound knowledge of how cells interact with lectin-coated surfaces is essential.

Cell adhesion to any surface can be divided into three phases, based on the duration of the contact [44,45]: (i) the initial phase (<1 min) dominated by adhesive contacts formed, (ii) the continuous spreading phase (from 1 min to several hours), and (iii) steady-state phase (typically after 24 h). Depending on the phase, various approaches can be applied to assess the adhesive properties of cells adhering to substrates [34,46]. Most methods characterize cell adhesion based on the number of attached cells or by quantifying the cell spreading area. With the development of nanoscale methods, the adhesion strength at the single-cell level can be quantified, for example, by single-cell force spectroscopy (SCFS), which measures the adhesion via quantification of work during the physical detachment of a cell from the surface [47].

Here, we focus on the interaction of bladder cancer cells with lectins DBA, LCA, PHA-L, and WGA. The selection of these lectins was based on their affinity to specific glycans combined with literature data showing potential differences in their expression in non-malignant cell cancer of ureter (HCV29 cells) and two grade 3 urothelial cell carcinomas (UCCs), i.e., HT1376 and T24 cells [5,10,21,48]. To understand the dynamic nature of cell adhesion, we evaluate the adhesiveness of bladder cancer cells dependent on spreading time from the initial to the steady-state phase on lectin-coated surfaces. The results allow us to trace how cell adhesion changes as a function of lectin and cell types. The ultimate goal was to verify whether abnormal glycosylation can be applied as a bio-marker of urothelial cancers.

## 2. Results

### 2.1. Cell Adhesion to Lectin-Coated Surfaces—From Initial Adhesion to Steady-State Phase

**Figure 1:**
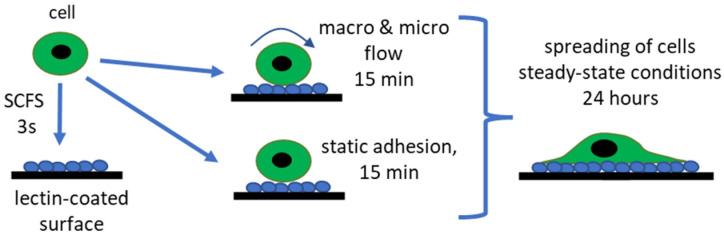
Timescale of cell adhesion, including identification of various methods applied to quantify the adhesive properties of cells.

The cell attachment to the lectin-coated surfaces begins with the formation of individual bonds between a cell and the surface. This stage is assumed to last one minute at maximum. Afterward, the cell flattens and spreads, reaching the steady-state phase, which takes minutes to hours [44,45]. Following this, we identify differences in the adhesion of bladder cells to lectin-coated surfaces using various methods to measure the adhesive properties of cells at various time scales (Figure 1).

We quantify cell adhesion to lectin-coated surfaces following the timescale of cells spreading. SCFS measures the adhesive properties at the initial adhesion stage when individual bonds are formed (time < 1 min). Considering the potential application of lectin-coated surfaces to collect cells with specific glycans on the surface, we decided to quantify the adhesion by counting the cell number after 15 min (during the spreading state) in static and flow conditions (in Ibidi and custom-designed microfluidic channels). To complete the story, cells at steady-state spreading (after 24 h), were quantified by calculating the spreading surface area.

### 2.2. Glycans Recognized by DBA, WGA, LCA, and PHA-L Are Detected in Bladder Cancer Cells

The intrinsic feature of lectins is to recognize the specific glycan types on the surface of living cells (Table 1).

**Figure 2:**
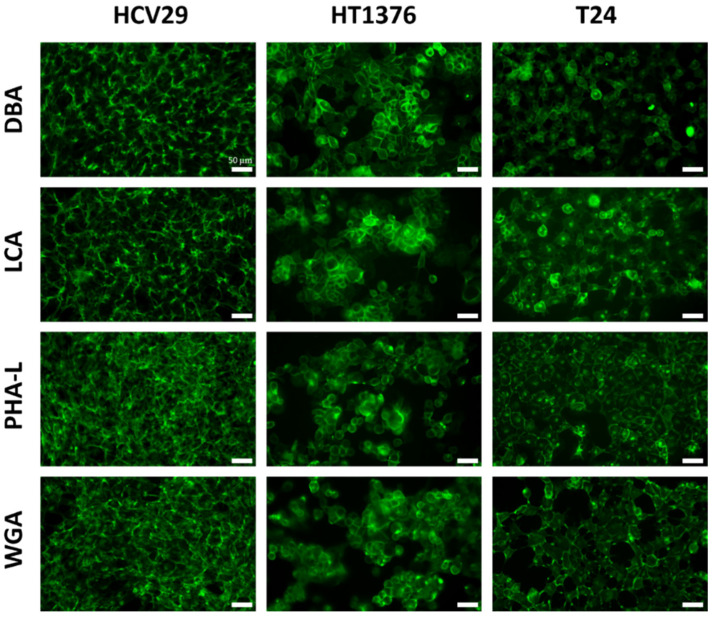
Fluorescence images of bladder cancer (HCV29, HT1376, T24) cells stained with FITC-conjugated lectins (green). Cells were incubated for 15 min with the selected lectins (DBA, LCA, PHA-L, WGA), fixed with 3.7% paraformaldehyde, and visualized by epifluorescence microscopy. Scale bars = 50 µm.

To verify the presence of lectins that attach to glycans on the surface of living bladder cancer cells, lectins labeled with fluorescein isothiocyanate (FITC, Figure 2) were used. The results show that fluorescently labeled lectins are uniformly distributed over the whole cells, with brighter fluorescence around the cell edge corresponding to the distribution of surface glycans they attach to. For the T24 cells, lectins are also observed in the nuclear region, which can be correlated to lectin uptake and their role in inducing caspase-dependent apoptotic response [50]. The presence of glycans recognized by DBA, LCA, PHA-L, and WGA lectins observed here agrees with published data [10,19].

In summary, fluorescent lectins recognizing specific glycans were evenly distributed on the surface of bladder cancer cells regardless of their type, i.e., non-malignant HCV29, carcinoma HT1376, or transitional cell carcinoma T24 cells.

### 2.3. WGA and PHA-L Coatings Differentiate Bladder Cancer Cells at the Initial Stage of Cell Adhesion

**Figure 3:**
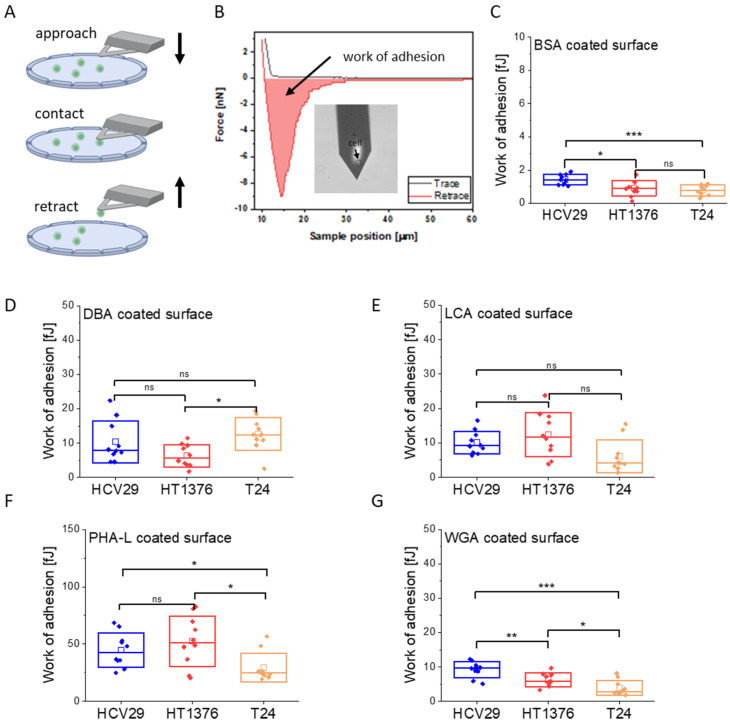
Adhesion of single bladder cancer cells (HCV29, HT1376, T24) to lectin-coated (DBA, LCA, PHA-L, WGA) surface at the initial stage of cell adhesion (contact time 3 s). (**A**) Schematic illustration of the cell probe preparation. (**B**) An example of a force - distance curve recorded for HCV29 cell probed against a PHA-L-coated surface, accompanied by optical images of such probe (insert). The integral of the retract curves for negative force from the contact point to the last detachment event denotes the work of adhesion—work needed to detach a single cell from a surface. (**C**–**E**) Boxplots showing the work of adhesion needed to detach single cells from the lectin-coated surface obtained for BSA (**C**, used here as a control), DBA (**D**), LCA (**E**), PHA-L (**F**), and WGA (**G**) coated surfaces. Each point denotes a mean of the work of adhesion calculated for data acquired with one cell probe. Box plots represent a median (line), a mean (open square), and a standard deviation (box). Statistical analysis was performed using the Mann—Whitney test. Statistical significance was obtained at the significance level of 0.05 (ns—no significant difference, * p < 0.05, ** p < 0.01, *** p < 0.001).

Having confirmed that all glycans recognized by lectins are present on the cell surface, SCFS was used to determine the initial cell adhesion to the lectin surface. The adhesion of cells to any surface starts with the formation of adhesive sites on the cell surface, typically occurring within the first minute [44]. By applying SCFS, we mimic the attachment of a single cell to a lectin-coated surface within a time scale of 3 s. This method brings a cell into contact, followed by its detachment during withdrawal (Figure 3A,B).

The magnitude of the adhesion between a single bladder cell and the lectin-coated surface was quantified by analysis of recorded force curves (at the approach and retract speed of 8 µm/s). The surface coated with bovine serum albumin (BSA) was used to assess the level of non-specific adhesion for bladder cancer cells due to the weak interaction of cells with BSA (Figure 3C).

A mean value of the work of adhesion was calculated based on an analysis of individual force-retraction curves (c.a. 100 force curves, Table 2). This was conducted for each cell-functionalized cantilever to obtain fully independent data. The final adhesion value was calculated as a mean of values obtained from 10 cell probes. The obtained results show lectin and cell type-dependent adhesion (Figure 3C–G). The smallest work of adhesion was observed for cells interacting with the BSA-coated surface (Figure 3C) and determined to be in the range of 0.78 fJ to 1.42 fJ, depending on the cell type. For the adhesion toward the BSA-coated surface, the non-malignant HCV29 and the transitional cell cancer T24 cells showed the largest and smallest adhesion, respectively. The adhesion was much larger for cells interacting with lectin-coated surfaces (Figure 3D–G). Among the lectin-coated surfaces, adhesion was the largest for PHA-L-coated surfaces regardless of the cell type. It was 44.8 ± 15.0 fJ for non-malignant HCV29 cells, 52.5 ± 21.9 fJ, and 29.5 ± 12.5 fJ for cancerous HT1376 and T24 cells, respectively. These results inversely correlate with previous measurements of single-molecule interactions between a PHA-L-coated cantilever and glycans on the surface of living T24 cells [5]. The unbinding force of an individual complex formed between PHA-L and N-acetyl-α-D-glucosamine (GlcNAc) was 3 times larger in T24 than in HCV29 cells. It could indicate that the surface density of GlcNAc glycans on the cell surface is lower than in the case of HCV29 cells. The adhesion work of bladder cells to other lectins is around ~10 fJ. However, only statistically significant differences were observed among cells interacting with the WGA-coated surface, where the work of adhesion was 9.24 ± 2.29 fJ, 6.25 ± 2.04 fJ, and 3.88 ± 2.14 fJ for HCV29, HT1376, and T24 cells, respectively. This indicates that WGA might be applied to differentiate between bladder cancer cell lines. The adhesion of HCV29/T24 and HT1376/T24 cells against PHA-L-coated surfaces shows a statistical difference. The use of other lectins (i.e., DBA and LCA) to differentiate among cell types is less obvious as the differences are much smaller. In conclusion, the SCFS results select WGA and PHA-L as potential surface coatings that could be applied to identify adhesion-related changes in bladder cancer cells at the initial stage of cell adhesion.

### 2.4. Static Adhesion Showed Distinct Adhesiveness of Bladder Cells to PHA-L and WGA Coatings

**Figure 4:**
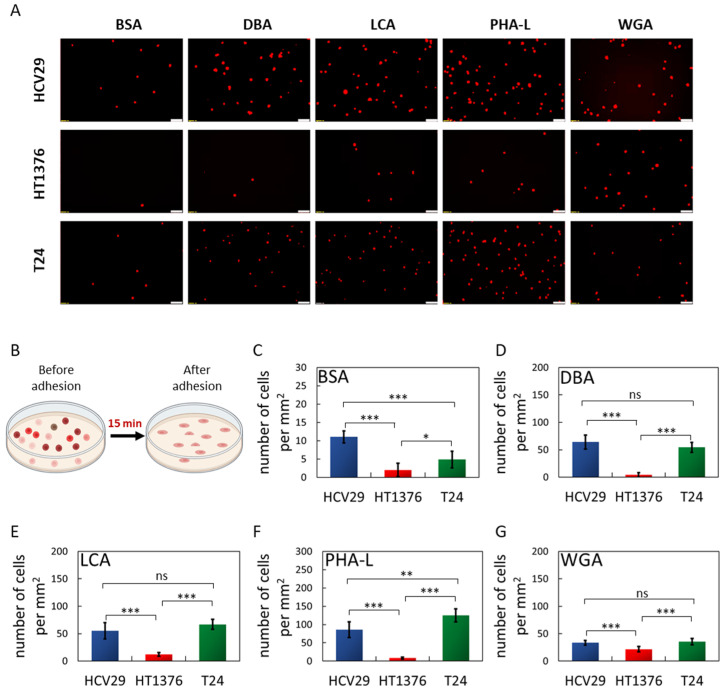
Adhesion of bladder cancer cells to lectin-coated surfaces in static conditions. (**A**) Representative fluorescence images of the bladder cancer cells labeled by Cell Tracker Red, attached to lectin-coated surfaces. Scale bar = 100 µm. (**B**) The bladder cancer cells were added to 24-well plates whose bottom surface was coated with either BSA or specific lectins for 15 min. After this time, the number of cells was counted and represented as a mean ± SD of 3 independent experiments. The surface was coated with BSA (**C**, control), DBA (**D**), LCA (**E**), PHA-L (**F**), or WGA (**G**). Statistical analysis was performed using one-way ANOVA with Tukey’s post-test (ns—no significant difference, * p < 0.05, ** p < 0.01, *** p < 0.001).

Following the initial cellular attachment to the surface, cells start to spread, which takes place on a timescale from minutes to hours. This phase is characterized by an incremental increase in the contact area with the substrate. Such spreading is accompanied by the formation of new adhesive sites and reorganization of the cell interior [51]. Therefore, studying cells in this phase requires a specific time to be set. Our next step estimated the number of cells attached after 15 min of cell spreading (Figure 4, Supplementary Table S1). Two controls were applied, an uncoated glass surface (Supplementary Figure S1) and a BSA-coated surface (Figure 4). The observations of only a few cells attached to the BSA-coated surfaces (Figure 4A,C) agree with the small work of adhesion obtained by SCFS. The cell surface densities on the BSA-coated surfaces varied with cell type, being 11.06 ± 1.63 cells/mm^2^ for HCV29 cells, 2.01 ± 1.84 cells/mm^2^ for HT1376 cells, and 4.88 ± 2.26 cells/mm^2^ for T24 cells, respectively. The cell surface density increases depending on the lectin and cell for the lectin-coated surfaces. For non-malignant HCV29 cells, the largest density of cells was observed on the PHA-L-coated surface (86.2 ± 21.5 cells/mm^2^), while the lowest was for the WGA-coated surface (33.3 ± 4.3 cells/mm^2^). The surface density of cells adhered to DBA- and LCA-coated surfaces was 50–60 cells/mm^2^, similar to that observed for attachment to an uncoated glass surface (Supplementary Figure S1). The density of carcinoma HT1376 cells was always the smallest compared to the observation for the other two cell lines. It varies between 4 to 22 cells/mm^2^ for the DBA- and WGA-coated surfaces. The obtained data showed that HT1376 cells adhere poorly to DBA (4.02 ± 1.42 cells/mm^2^) and PHA-L-coated surfaces (8.33 ± 2.59 cells/mm^2^) and stick better to WGA-coated surfaces (21.6 ± 4.8 cells/mm^2^). Carcinoma T24 cells stick better to PHA-L-coated surfaces (125.3 ± 17.8 cells/mm^2^) and poorly to WGA-coated surfaces (35.3 ± 5.8 cells/mm^2^). Their attachment to DBA and LCA lectins was akin to that observed for non-malignant HCV29 cells (i.e., 50–60 cells per mm^2^, no statistical difference).

Overall, the largest cell density was observed for non-malignant HCV29 and cancerous T24 cells on PHA-L-coated surfaces, while the lowest was observed for WGA-coated ones. The opposite trend was observed for carcinoma HT1376 cells. They adhere better to WGA than PHA-L-coated surfaces.

### 2.5. Applying Low Shear Stress Changes the Adhesion of Bladder Cells to Lectin-Coated Surfaces

**Figure 5:**
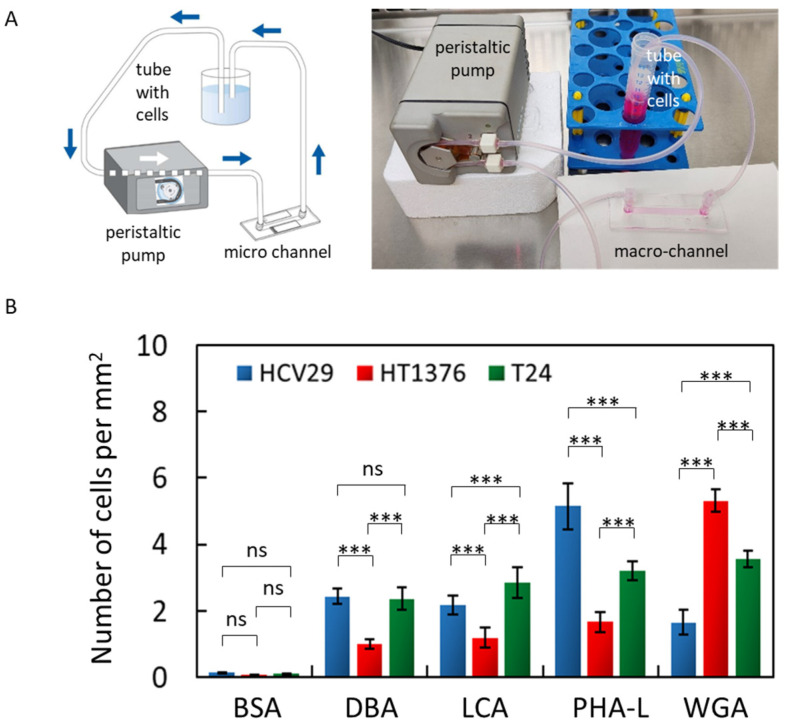
(**A**) Schematic illustration and a photo of the self-constructed macro flow system. It consists of a peristaltic pump generating a flow within the volumetric flow (0.3–2.8 mL/min), test tubes (Sarstedt) filled with cells, and a channel (µ-Slide I Luer, height 400 µm, width 5000 µm and length 50 mm) whose surface was coated with the specific lectin. (**B**) The number of bladder cancer cells per mm^2^ adhered to lectin-coated surfaces in the applied wall shear stress (0.0373 Pa). The results are presented as a mean ± SD of three independent experiments. Statistical analysis was performed using one-way ANOVA with Tukey’s post-test (ns—no significant difference, *** p < 0.001).

Intuitively, a transition from static to flow conditions can change the adhesive properties of cells due to the effect of wall shear stress [52] (Supplementary Note S1). First, the adhesiveness of bladder cancer cells was evaluated under flow conditions using a home-built flow system that provides small wall shear stress. The applied volumetric flow rate of the culture medium [53] corresponds to a wall shear stress of 0.0373 Pa (at 37 °C, Supplementary Table S2). The results are expressed as the number of cells per mm^2^ (Figure 5B, Supplementary Table S3), similar to those reported for the static adhesion assay.

For the applied flow conditions with wall shear stress equaled to 0.0373 Pa, bladder cancer cells practically do not attach to the BSA-coated surfaces (0.13 ± 0.02 cells/mm^2^, 0.07 ± 0.01 cells/mm^2^, and 0.10 ± 0.01 cells/mm^2^ for non-malignant HCV29, and cancerous HT137 and T24 cells, respectively). Coating the Ibidi-channels with lectins increased the numbered of adhered cells depending on the lectin and cell type. For HCV29 cells, the highest cell surface density was observed for the PHA-L-coated surface (5.15 ± 0.70 cells/mm^2^) and the smallest for the WGA-coated surface (1.66 ± 0.38 cells/mm^2^), which agrees with static adhesion assay showing similar dependency. For HT1376 cells, the largest surface density was observed for WGA-coated surface, 5.31 ± 0.34 cells/mm^2^, and the lowest for DBA-, LCA-, and PHA-L-coated surfaces, all observed at a similar level of 1–2 cells/mm^2^. These trends align with those observed in the static adhesion assay, analogously for non-malignant HCV29 cells. The results for T24 cells were different, and no clear correlation with the results from the static adhesion assay was found. T24 cells adhere best to PHA-L and WGA in a comparable way, i.e., 3–4 cells/mm^2^, and with slightly less surface density than DBA- and LCA-coated surface (2–3 cells/mm^2^).

These results show that adhesive properties are not constant and change depending on the conditions in which these cells are placed. Simultaneously, these results suggest that T24 cells are more adaptive to the surrounding environment due to their invasive character [54] than other bladder cancer cells. Despite the origin of bladder cancer cell adhesion, these results also indicate that PHA-L and WGA are better candidates to attract specific cell types among the four studied lectins.

### 2.6. Applying High Shear Stress Induces Similar Changes Observed under Low Shear Stress

**Figure 6:**
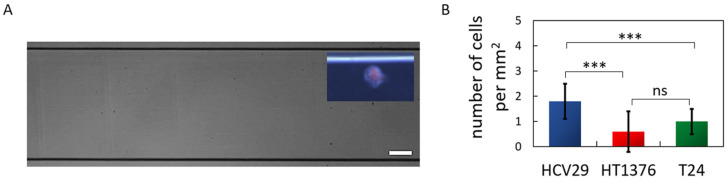
The effect of high wall shear stress (0.185 Pa) on bladder cancer cell adhesion to PHA-L-coated channel. (**A**) Microfluidic channel (scale bar 100 µm). Inset: Fluorescently marked HCV29 cells adhered to the glass bottom of the PHA-L-coated surface (40× objective lens). (**B**) The number of bladder cancer cells per mm^2^ adhered to PHA-L-coated surfaces (±SD) under the applied wall shear stress of 0.185 Pa. Statistical analysis was performed using one-way ANOVA with Tukey’s post-test (ns—no significant difference, *** *p* < 0.001).

The effect of higher wall shear stress on cell adhesion was studied using smaller channels. Therefore, a microfluidic experiment was performed for all bladder cancer cell lines studied with a PHA-L-coated surface (Figure 6). The wall shear stress [55] generated in the microfluidics channel was 0.185 Pa (Supplementary Table S2). The microfluidic set-up was designed to complement the Ibidi-flow system by preserving the average flow velocity and cross-sectional dimensions of the channel. Hence the main variation was the wall shear stress and the channel surface area (Supplementary Figure S2).

Increasing the shear stress to 0.185 Pa (microfluidic channel) yielded similar trends in the differences among bladder cell types adhering to PHA-L-coated surface as those observed for the Ibidi channels, where non-malignant HCV29 cells showed a higher preference for PHA-L-coated surface adhesion than HT1376 and T24 cells (Figure 6B). The low number of observed adhesion events was consistent with results from the literature [38,56] and the Ibidi channel, as the cells did not adhere evenly throughout the channel. Thus, the reduction of the channel surface area (from 250 to 15 µm^2^) and increase of the wall shear stress (from 0.0373 to 0.185 Pa) leads to a reduction in the number of observed adhered cells.

### 2.7. The Spreading of Bladder Cancer Cells Is Sensitive to Lectin Coatings

**Figure 7:**
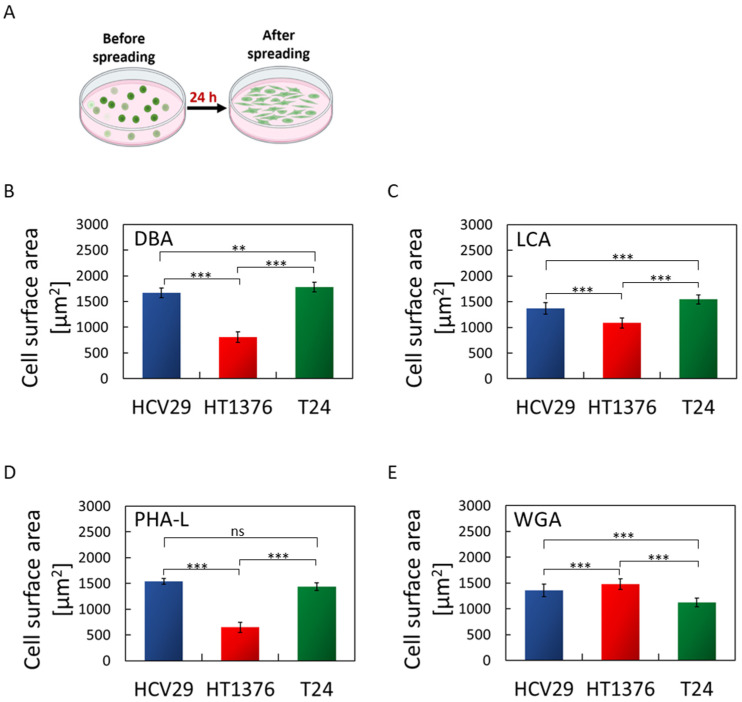
Spreading of bladder cancer cells on lectin-modified surfaces quantified as the average surface area per spread cell. Results are presented as a mean ± standard deviation from approx. 300–600 cells, an average of 3 independent repetitions. (**A**) Scheme of the experiment used to determine single-cell spreading area based on fluorescence images of actin filaments and cellular nuclei. (**B**–**E**) Spreading capability of HCV29, HT1376, and T24 cells on the lectin-coated surfaces (**B**) DBA, (**C**) LCA, (**D**) PHA-L, and (**E**) WGA. Student’s *t*-test (at the significance level of 0.05) was applied to statistically verify the obtained results (one-way ANOVA with Tukey’s post-test, ns—no significant difference, ** p < 0.01, *** p < 0.001).

Cells reach a maximum spreading area after approximately 24 h [57]. In this steady-state phase, cells possess a fully organized cytoskeleton structure with stable adhesion. Cells with larger spreading areas are expected to show stronger attachment to the substrate, and a larger cell adhesion [34]. Thus, we elaborate on how the chosen bladder cancer cells spread on surfaces coated with lectins. Images of fluorescently labeled F-actin were collected for each cell line culture on glass and lectin-coated surfaces (Supplementary Figure S3). Each cell line in our experiments possesses characteristic morphology, as evident from cultures on a bare glass surface. Non-malignant HCV29 are elongated, spindle-like cells with thick visible actin bundles spanning a cell body. They grow as non-confluent cells in a fibroblast-like manner (long axis ~130 µm, short axis ~33 µm, *n* = 20 cells). Carcinoma HT1376 cells are round (d = ~38 µm, *n* = 20 cells) and tend to grow in clusters, although individual cells are also present. No thick actin bundles were observed in these cells, similar to that observed for HCV29 cells. F-actin is rather uniformly distributed over the cell. Carcinoma T24 cells, like HT1376, were also observed with round morphologies (*d* = ~35 µm, *n* = 20 cells), but seem to be better spread, and the thick actin bundles are visible like in HCV29 cells. Next, the cells were cultured on lectin-coated surfaces to verify whether and how the presence of lectins affects the morphology of these cells. Fluorescent images show that only non-malignant HCV29 cells were unaffected by lectin coating. The morphology of T24 cells, changed by increase in size and tendency to cluster, was observed in response to the lectin type. The largest morphological change of the HT1376 cells was observed when grown on the surface coated with PHA-L. This leads us to conclude that cancer cells are more sensitive to lectin coatings when grown on adherent surfaces than non-malignant HCV29 cells. Images of fluorescently labeled F-actin were applied to quantify the cell spreading by calculating the effective surface area of a spread cell (Figure 7).

Non-malignant HCV29 cells were observed with the largest cell surface area on PHA-L-coated surface (average 1540 ± 60 µm^2^/cell) and the smallest when adhering to WGA-coated surfaces (1360 ± 120 µm^2^/cell). Cancerous T24 cells show a similar dependence in spreading area for these two lectin-coated surfaces. The obtained values of the effective surface area were 1440 ± 70 µm^2^/cell and 1130 ± 90 µm^2^/cell for PHA-L- and WGA-coated surfaces. Carcinoma HT1376 behaves differently. These cells spread better on WGA-coated surfaces (1480 ± 100 µm^2^/cell) and worse when PHA-L was applied as a coating (650 ± 100 µm^2^/cell). The results are consistent with the number of cells attached in static and flow assays pointing to PHA-L and WGA lectins as a potential coating that can be applied to identify the specific type of bladder cancer cells.

## 3. Discussion

The gathered data have convincingly described that cells that acquired a neoplastic phenotype alter their adhesive properties depending on the stage of cancer progression [2,3,4]. Among others, changes in the structure and the expression of glycans (i.e., abnormal glycosylation) present on the surface of the cells may play a role in the metastasis process, in which glycans may act as receptors bound to ECM [58]. However, although much is known about cancer-related changes in glycosylation patterns, the specificity of the glycan–lectin binding is not fully elaborated, especially in static and dynamic conditions.

We focused on bladder cancer cell lines as this cancer is frequently diagnosed globally, and mortality highly depends on the cancer stage [59,60]. Although about 75% of cases are non-invasive cancer, there is still a high risk of disease recurrence [61,62]. Treatment of patients with bladder cancer that has not expanded beyond the urinary tract epithelium involves transurethral resection of the tumor, while invasive tumors usually need a combination of cystectomy, radiation therapy, and/or chemotherapy. On the other hand, approximately 60–70% of non-muscle invasive bladder cancer recurs and requires clinical re-intervention [63]. Despite the current therapeutic approaches, a major concern is the potential tumor resistance and the low response rate [64]. It is still emerging for modern oncology to develop rapid, accurate, and effective tools for early detection, detailed diagnosis, and staging of bladder cancers. Aside from the precise determination of the cancer type, such tools are essential for defining adequate therapy, which reduces the risk of recurrence, lowers mortality rates, and improves patients’ quality of life. It seems that microfluidic devices based on differences in adhesiveness can be used to identify cancer cells. Previous reports have demonstrated the effectiveness of such an approach in differentiating between various types of cancers, including breast and colon cancers [65,66].

Cells cannot adhere to the substrate if no specific ligands are present on the material surface with which the cell adhesion molecules can interact [67]. When a cell comes into contact with a surface, short-range physicochemical interactions (van der Waals, Coulomb, and steric forces) are formed. These bonds initiate adhesive contact formation, allowing the cell to spread [68]. If the surface properties are favorable, the second phase begins, where the cell flattens and takes on a characteristic shape. This phase is dynamic due to the continuous remodeling of the actin cytoskeleton and the switching of adhesive contacts to focal contacts (called focal adhesions). In the third and last phase, the cytoskeleton is well-organized with matured focal adhesions containing integrins, primary cell surface receptors, that bind to the ECM [44,68]. As glycans are attached to proteins or lipids [25,36], the observed alterations in glycosylation level indicate simultaneous changes in integrins, cadherins, or other surface expression receptors responsible for cell-to-cell or cell-to-ECM interactions [58]. Intuitively, such a process should be accompanied by dynamic changes in cell adhesion. Thus, by applying various methods to trace the adhesiveness of bladder cancer cells, as we have demonstrated here, one can evaluate to what extent such changes depend on the culture conditions and duration of spreading.

The initial phase of cell adhesion was assessed by SCFS [47,69]. The SCFS showed that different forces/energy must be applied to detach the various bladder cancer cells from the surface functionalized with selected lectins. The results showed that the maximum work of adhesion was obtained for the PHA-L-coated surface. Obtained work of adhesion for other lectins, i.e., DBA, LCA, and WGA, was remarkably similar. The SCFS measurements are limited to 3 s of contact time between the cell and the surface. Notably, the adhesion of bladder cancer cells to a PHA-L-coated surface, especially in the case of HCV29 and T24 cells, correlated with single molecule data on the unbinding properties of the individual complex [5]. Thus, the adhesion work strongly depends on the contact area (defined by the load force and cell diameter) and how many adhesive contacts (and how strong they are) can be formed within this space and time [70].

Other applied methods rely on counting the number of cells attached to the surface or estimation of changes in cell spreading area on the surface. The adhesion is dependent on the same factors as mentioned above. Within the contact area, the adhesion originates from multiple specific and non-specific interactions, and it is impossible to identify how many glycans bind to specific lectins and the contribution of other non-specific elements. It is possible to state that the adhesion between the cell and a particular lectin deposited on the surface is dominated by glycans binding to this lectin. Therefore, the observed differences among the three studied bladder cancer cells stem from the variability in the density (number of glycans/surface) and/or distributions of specific glycans. Thus, one of the questions we asked was how adhesive properties translate among various methods. Can we get similar results? The obtained results revealed that qualitative relationships are similar and not dependent on the methods used to assess the adhesive properties of cells. However, there is a strong dependence on the cell type. Non-malignant HCV29 cells showed higher adhesion work, the number of cells per mm^2^ attached to the surface in static and flow conditions, and a larger surface area of a spread cell for the PHA-L-coated surfaces than for the WGA-coated ones. For cancerous HT1376 and T24 cells, such a relation is not fully preserved. For HT1376 cells, the results showed better adherence to WGA-coated surfaces, which is visible at the later phase of cell spreading (i.e., static and flow conditions, spreading surface area), but at the initial adhesion phase, the binding to WGA is weaker than to PHA-L. For T24 cells, stronger interaction with the PHA-L-coated surface was observed in static conditions and at the steady-state phase, but not in flow conditions and at the initial phase of the adhesion (in the SCFS data). These results may suggest cancer cell adaptability, which generally is directed towards enhancing invasiveness.

The ultimate goal of the presented study was to identify which of the studied lectins can be applied as a surface coating for the selective capturing of cancer cells. Our findings show that, regardless of the applied method to assess the adhesion of cells, two lectins can be applied as a surface coating as potential candidates for capturing cells with altered glycosylation patterns. These lectins are PHA-L and WGA, interacting differently for non-malignant and cancerous cells. However, different lectins have to be chosen depending on the cancer type. The results show that PHA-L and WGA lectins can be applied to differentiate between non-malignant HCV29 and cancerous HT1376 cells, while PHA-L is more suitable for carcinoma T24 cells. These findings strongly support the hypothesis that plant lectins can be used as substrates for the selective capture and differentiation between normal and cancerous cells, which, together with altered cell deformability, could improve microfluidics-based methods. From the clinical point of view, there is still a need to develop novel, low-cost, and effective biomarkers [71]. Distinct molecules can be found in the patient’s urine (exfoliated tumor cells, proteins, genes, metabolites, or extracellular vesicles (EVs)). Biosensors, prepared based on lectins, could potentially selectively capture such exfoliated tumor cells or even extracellular vesicles in a non-label and quantitative manner.

## 4. Materials and Methods

### 4.1. Lectins

Commercially available plant lectins, i.e., *Dolichos Biflorus Agglutinin* (DBA), *Lens Culinaris Agglutinin* (LCA), *Phaseolus vulgaris* (PHA-L), and *Wheat Germ Agglutinin* (WGA) were purchased in Vector Laboratories (Biokom, Janki, Poland) and used in all experiments. Concanavalin A (Con A) from *Canavalia ensiformis* was purchased from Sigma-Aldrich (Poznań, Poland). Lectins were dissolved in phosphate-buffered saline (PBS, P4417, Sigma-Aldrich, Poznań, Poland) at 0.1 mg/mL concentration or in deionized water (0.1 mg/mL).

### 4.2. Cell Cultures

The HCV29 (non-malignant cell cancer of the ureter; a kind gift from Prof. Piotr Laidler from the Chair of Medical Biochemistry, Collegium Medicum Jagiellonian University, Kraków, Poland; [72]) and T24 (urothelial cell carcinoma, older name—transitional cell carcinoma, grade 3, ATCC, LGC Standards) cells were cultured in Roswell Park Memorial Institute Medium 1640 (RPMI 1640, R8758, Sigma-Aldrich, Pozań, Poland) supplemented with 10% fetal bovine serum (FBS, Sigma-Aldrich, Poznań, Poland). HT1376 (urothelial cell carcinoma, grade 3, ATCC, LGC Standards) cells were grown in Eagle’s Minimum Essential Medium (EMEM, LGC Standards) supplemented with 10% FBS. They were cultured in the CO_2_ incubator (Nuaire) at 37 °C in an atmosphere of 5% CO_2_ and 95% humidity. Cells were cultured in 25 cm^2^ culture flasks (TPP, Genos, Poland). All cell lines were routinely observed under an optical microscope (Olympus). The cells were passaged twice a week. Cell line authentication was performed by using the FTA Sample Collection Kit for Human Cell Authentication Service (LGC Standards).

### 4.3. Cell Size Determination

Cell cultured in 25 cm^2^ culture flasks were washed 2 times with PBS, followed by adding 1 mL of 0.25% ethylenediaminetetraacetic acid (EDTA)-trypsin (Sigma-Aldrich, Poznań, Poland) and placed in the CO_2_ incubator at 37 °C. After 5 min, a culture medium supplemented with 10% FBS was added to the culture flasks and gently mixed. Cells were transferred to 15 mL tubes and centrifuged at 1800 rpm for 4 min at room temperature (RT), which formed a cell pellet at the bottom of the tube. Next, the supernatant was replaced with fresh culture medium supplemented with 10% FBS and gently mixed to obtain cell suspension. The number of cells was obtained by counting them in the Burker chamber (Marienfeld, Germany). Samples for the optical microscope were prepared in a 6-well plate (TPP, Genos, Poland). A 0.5 mL aliquot of the cell suspension containing 150,000 cells/mL was added to 2 mL of culture medium, filling each well. A plate was placed in the optical microscope (Olympus, type CKX53). After 5 min, phase-contrast images were collected (5 images per cell line) at 20× magnification. The diameter of single cells was determined using Image J software (version 1.53k, https://imagej.nih.gov/ij/, 6 July 2021). The data are expressed as a mean ± standard deviation from at least 100 randomly chosen cells. The diameter of undeformed, round cells was 12.6 ± 1.6 µm, 11.7 ± 1.8 µm, and 7.9 ± 0.9 µm for HCV29, HT1376, and T24 cells, respectively.

### 4.4. Preparing Surfaces for SCFS

SCFS measurements were carried out in Petri dishes where the surfaces had been coated with the given lectin (DBA, LCA, PHA-L, or WGA) or with BSA (Sigma-Aldrich, Poznań, Poland), all dissolved in deionized water (0.1 mg/mL). BSA was used as a control protein to which cells do not adhere. The Petri dish surfaces were functionalized with lectins by incubating them with solutions of the respective lectins (DBA, LCA, PHA-L, WGA) or BSA for one hour at RT. Subsequently, the residual solution was removed, and surfaces were washed three times in PBS.

### 4.5. Preparing Cell Probes for SCFS

SCFS measurements were realized based on the commercially available tipless cantilevers (Arrow-TL, nominal spring constant of 0.03 N/m, NanoWorld) used to create cell probes, i.e., tipless cantilever with a single living cell attached to a free end (one cantilever with one cell). Cell attachment to cantilevers was realized by first cleaning and activating cantilevers with oxygen plasma (Zepto 1 device, Diener Electronics GmbH) for 1 min at the maximum power of 100 W. Next, they were functionalized with Con A) by immersing them in a solution of 2 mg/mL Con A in PBS for one hour at RT. The cantilevers were subsequently washed three times in PBS to remove unbound proteins. The Con A -coated cantilevers were calibrated with respect to the spring constant (0.081 ± 0.017 N/m, n = 10) and photodetector sensitivity (68.4 ± 6.5 nm/V).

To attach a single cell to the free end of the tipless cantilever, the trypsinized cell suspension of HCV29, HT1376, or T24 cells was transferred to the Petri dish, previously coated with a specific lectin or BSA. Next, the Con A-functionalized cantilever was positioned over the chosen cell and approached the cell surface at a velocity of 5 µm/s. When contact between the cantilever and the cell surface was established, the cantilever pressed the cell with a force of 3 nN for 5 s. Next, the cantilever with a captured cell was slowly withdrawn (5 µm/s) for a few µm above the surface and left for 15 min to allow the cell to firmly attach to the cantilever surface. The freshly prepared cell probes were kept in PBS and were immediately used in the SCFS experiments.

### 4.6. SCFS

All SCFS measurements were conducted using an atomic force microscope (AFM) equipped with a CellHesion head (Bruker-JPK Instruments, Berlin, Germany). Force curves, i.e., dependencies between cantilever deflection and relative sample position, were collected with a specific force probe (HCV29 or HT1376, or T24 cell probes were used). The force curves were recorded at a 10 × 10 points grid over the lectin or BSA-coated surface of 10 μm × 10 μm. A total of about 100 force curves were acquired for each cell probe. The force curves were automatically recorded using an approach and retract speed of 8 μm/s. The contact time was 3 s. The total number of force curves collected for each cell-lectin type combination is summarized (Table 2).

The work of adhesion was calculated from experimentally collected force curves to quantify the adhesion strength of a cell to a specific protein (lectin or BSA). It was quantified by determining the integral of the force retraction curve under the adhesive part (force < 0) from the contact point to the return of the cantilever to the baseline using JPK Data Processing software (Bruker-JPK Instruments).

### 4.7. Labeling Glycans on the Cell Surface

Cells were seeded in a 6-well plate at 10^5^ cells/well density. The cells were grown in the corresponding culture medium for 24 h at 37 °C in the 5% CO_2_ atmosphere and 95% humidity. Then, the culture medium was replaced with a fresh one. Next, fluorescent FITC conjugated lectins (FITC-DBA, FITC-LCA, FITC-PHA-L, and FITC-WGA; Vector Laboratories; Biokom, Janki, Poland) were added to each well at a concentration of 5 µg/mL and incubated at 37 °C for 30 min. The medium was subsequently removed, and cells were washed three times in PBS to eliminate unbound lectins. Then, cells were fixed with 3.7% paraformaldehyde in PBS (20 min at RT) and washed 3 times with PBS. A fluorescence microscope was used to visualize the lectins labeled bladder cancer cells. The experiments were conducted three times.

### 4.8. F-Actin Visualization

Cells were cultured on 6-well plates coated with lectins (DBA, LCA, PHA-L, and WGA) in 2 mL corresponding culture medium supplemented with 10% heat-inactivated FBS (at 37 °C, 5% CO_2_, and 95% humidity atmosphere for 24 h). The following protocol was applied to fluorescently stained F-actin. Cells were washed in PBS (3 times, each time for 2 min), followed by fixation (3.7% paraformaldehyde solution in PBS, 20 min incubation at RT), rinsing (PBS, 3 times, each 2 min duration), permeabilization (a cold 0.2% Triton X-100 (Sigma-Aldrich, Poznań, Poland) for 5 min at 4 °C), and subsequent washing with PBS. To visualize the actin filaments, cells were incubated with phalloidin (0.033 µM in PBS, Molecular Probes, Thermo Fisher Scientific, Waltham, MA, USA) conjugated with Alexa Fluor 488 dye for 45 min at RT (in the dark). After washing the cells with PBS, the cell nucleus was counterstained using the Hoechst solution (1 µg/mL in PBS, Sigma-Aldrich, Poznań, Poland) for 15 min, followed by PBS rinsing. Such double-labeled cell samples were visualized using an epi-fluorescent microscope.

### 4.9. Preparing Cells for Static Adhesion Assay

Cells cultured in flasks were detached using a 0.25 % trypsin-EDTA (Sigma-Aldrich, Poznań, Poland) and centrifuged at 1800 rpm for 4 min. The supernatant was removed, while the cell pellet was dissolved in 4 mL of the culture medium. Cells were counted using a Bürker chamber. Cells were subsequently re-suspended at 10^6^ cells/mL in the culture medium containing 1 µg/mL of Cell Tracker Red dye (Invitrogen, Thermo Fisher Scientific, Waltham, MA, USA) and incubated for 30 min at 37 °C in the incubator providing the 5% CO_2_/95% air atmosphere. The cell suspension was subsequently centrifuged to obtain a fluorescently labeled cell pellet, the medium was removed, and cells were re-suspended in PBS and centrifuged again. This procedure was used to remove unbound fluorescent dye from the medium. The obtained cell pellet was suspended in a complete culture medium and used to assess cell adhesion to lectin-coated surfaces.

### 4.10. Adhesion Assay in Static Conditions

For static adhesion assay, 24-well plates were coated with a specific lectin (solution at 0.1 mg/mL lectin; 250 µL was added to each well), incubated for 2 h at RT, followed by removing the lectin solution, and drying the plates (20 min). As a control, the non-coated surface of the plate was used. Then, 5 × 10^4^ cells/mL were added to each well and incubated for 15 min at 37 °C, in the CO_2_ incubator. The non-adherent bladder cancer cells were removed by rinsing the wells three times with PBS. Adherent cells were fixed (with 3.7% paraformaldehyde solution in PBS for 20 min at RT) and washed with PBS, and fluorescent images of 3 randomly selected visual fields for every well were acquired using an Olympus IX83 fluorescence microscope. The static adhesion of bladder cancer cells was expressed by the number of cells in the fluorescent image 1100 µm × 702.6 µm. The experiment was repeated three times (3 images per repetition, for each repetition, a mean ± standard deviation was determined). The final results are presented as the number of cells per mm^2^ and expressed as a mean ± standard deviation calculated from each value obtained for a single repetition.

### 4.11. Preparing Cells for Flow-Based Adhesion

Cells cultured in flasks were detached using a 0.25% EDTA-trypsin solution. The suspension of detached cells was centrifuged at 1800 rpm for 4 min, and the supernatant was removed. Then, the cell pellet was dissolved in a culture medium supplemented with FBS. Cells were counted using a Bürker chamber. Next, cells were suspended at 15 × 10^4^ cells/mL in the culture medium containing 1 µg/mL fluorescent dye. HCV29 cells were labeled with Cell Tracker Blue (Invitrogen, Thermo Fisher Scientific, Waltham, MA, USA), HT1376 cells with Cell Tracker Red (Invitrogen, Thermo Fisher Scientific, Waltham, MA, USA), and T24 cells with calcein-acetoxymethyl (calcein-AM dye, green, BD Pharmigen, BD Biosciences, Franklin Lakes, NJ, USA). The cells were incubated with the fluorescent dye (30 min at 37 °C in the CO_2_ incubator), followed by rinsing 3 times in PBS, and re-suspended in an appropriate culture medium.

### 4.12. Adhesion Assay in Flow Conditions

For flow-based adhesion assay, the surface of the channels (Ibidi µ-Slide I Luer, ibidi GmbH, Gräfelfing, Germany; channel length 50 mm, width 5 mm, and height 400 µm) was coated with a specific lectin (0.1 mg/mL) for 2 h at RT, then dried and connected to the flow system. The fluorescently labeled cells were re-suspended in a 3 mL appropriate culture medium (RPMI 1640 for HCV29 and T24 cells and EMEM for HT1376 cells). A total volume of 9 mL of the cell suspension was placed in a tube (Saarstedt, Nümbrecht, Germany) and carefully vortexed. The tube with the cell suspension (5 × 10^4^ cells per mL) was connected to the flow system using connecting silicon tubes. Next, the peristaltic pump (P720, Instech Laboratories Inc., Plymouth Meeting, PA, USA; range of volumetric flow 0.3–2.8 mL/min) was switched on to establish a flow rate of 0.3 mL/min. In the applied Ibidi-channels, such a flow (at RT) generates wall shear stress of 0.0579 Pa (Supplementary Table S2) when the culture medium is used as a cell carrier fluid (for PBS, shear stress value is 0.0398 Pa, Supplementary Table S2). After 15 min, the flow was stopped, and the slide was rinsed with PBS buffer to remove non-adherent cells. The final results are presented as the number of cells per mm^2^ and expressed as a mean ± standard deviation calculated from each value obtained for a single repetition.

### 4.13. Epi-Fluorescent Microscopy

All images were recorded using an inverted optical microscope (Olympus IX83) equipped with a fluorescence module. A 100 W mercury lamp was applied to excite fluorescent dyes. A U-FBW filter (λ_exit_ = 460–495 nm, λ_emit_ = 510 nm) was used to collect fluorescent images of F-actin (Alexa Fluor 488, calcein-AM), while a U-FUW filter (λ_exit_ = 340–390 nm, λ_emit_ = 420 nm) was applied to record images of Hoechst-stained cell nucleus. Cell Tracker Blue CMAC and Cell Tracker Red CMTPX were collected with the use of U-FUW (λ_exit_ = 340–390 nm, λ_emit_ = 465 nm) and U-FGW (λ_exit_ = 530–550 nm, λ_emit_ = 575 nm) filters, respectively. Images were acquired using the ORCA-spark (Hamamatsu Photonics, Hamamatsu, Japan) digital camera, providing a 2.3 megapixel 1920 px × 1200 px image. Images were analyzed using Image J software (version 1.53k, https://imagej.nih.gov/ij/, 6 July 2021).

### 4.14. Determination of Surface Area of Spread Cells

To quantify the surface area of the spread cells, fluorescence images of cells with labeled F-actin and cell nuclei were analyzed as previously described [73]. Each image encompassed an area of 562.6 µm × 351.6 µm. In the first step, the surface area coated by cells was determined for each image, followed by counting the number of cell nuclei included within the recorded image (each nucleus denotes one single cell). Then, the surface area was divided by the number of cell nuclei, thus yielding the average surface area per cell. The experiments were conducted three times. In total, for each condition, 30 images were collected. The final value of the effective surface area for single cells was determined as a mean ± standard deviation from 300–600 cells.

### 4.15. Imaging Ibidi-Channel Surface after Flow Experiment

To estimate the number of cells attached to lectin-coated surfaces in the flow Ibidi-channels, a set of ~150 images was acquired (referred to here as a fluorescent scan). The average surface area of a single fluorescent scan (i.e., an image of the entire surface of the channel) equals 178 ± 29 mm^2^. Fluorescence scans were processed using ImageJ (version 1.53k, https://imagej.nih.gov/ij/, 6 July 2021). First, the Rolling Ball algorithm was applied to subtract the background noise containing repetitive non-cellular structures. The radius of the rolling ball sphere was set to 50.0 pixels. Then, the Threshold was applied to obtain binary masks, leaving only the signal from the cells attached to the surface. The number of cells was calculated using the Particle Analysis algorithm. Objects at the image boundaries were excluded from the analysis (Supplementary Note S2). Live/dead staining was conducted additionally to assess cell viability (Supplementary Note S3, Supplementary Figure S7).

### 4.16. Preparation of the Microfluidic Channels

The microfluidic channels were designed with a length of 3 cm, 50 µm height, and 500 µm width, maintaining the limiting aspect ratio of 1:10, which occurs for soft-lithography and PDMS (polydimethylsiloxane, Sylgard 184, Dow, Midland, TX, USA). The channels were further designed with one inlet and one outlet. Microfluidic channels were fabricated using photolithographic methods. First, a negative resist (SU-8 3050, micro resist technology, Berlin, Germany) was spun (Spin coater, Laurell Technologies, Landsdale, PA, USA) onto a silicon wafer and then soft-baked. The resist was then exposed to UV light (365 nm) using a maskless aligner (MLA100, Heidelberg-Instruments, Heidelberg, Germany) and, post-exposure baked at the appropriate temperature and duration. The mold was then developed using the solvent MrDev600 (microresist technology, Berlin, Germany), yielding the final master mold. Before applying soft lithography, the mold was silanized with trichloro(1H,1H,2H,2H-perfluorooctyl)silane (Sigma-Aldrich). PDMS was poured over the mold at a 1:10 (curing agent: elastomer) ratio and baked at 65 °C for 3 h. The microfluidic device was realized by removing the PDMS from the mold and punching inlet and outlet holes using a biopsy needle (1 mm), followed by plasma oxidation to seal the PDMS on a glass microscope slide.

### 4.17. Microfluidic Flow Experiment

The microfluidic channels were functionalized with PHA-L (0.1 mg/mL in PBS) and left for 2 h at RT before use. The cell solution used for the microfluidic device followed a similar protocol as for the flow in Ibidi-channels. However, instead of re-suspending cells in the culture medium for the final step, the microfluidic cell suspension used PBS achieving a final concentration of 10^5^ cells/mL. All cells were labeled with Cell Tracker Blue (Invitrogen, Thermo Fisher Scientific, Waltham, MA, USA). The suspension was left for 30 min at 37 °C before being vortexed for 2 min to increase the number of single cells in the solution and make it more homogeneous. The microfluidic device was flushed with PBS before the cell suspension was introduced. The adhesion experiments under flow were conducted with a flow rate of 200 µL/h for 60 min at 37 °C in a constant temperature room. The wall shear stress value equals 0.185 Pa (Supplementary Table S2). Afterward, the channels were carefully flushed with PBS (37 °C) to remove all non-adhered cells and immediately imaged by fluorescence microscopy (Nikon Eclipse Ti2; LED light source at 385 nm). The surface area of the microfluidic channels was 15 mm^2^.

### 4.18. Statistical Analysis

All experiments were repeated at least three times independently unless otherwise stated. Experimental data are presented as a mean ± standard deviation (SD). Statistical significance was estimated using a nonparametric Mann–Whitney test or one-way ANOVA with Tukey’s post-test, accessible in the OriginLab 2021 software. Notation: ns- not statistically different, * p < 0.05, ** p < 0.01, *** p < 0.001.

## 5. Conclusions

Glycans are important components of essential molecules such as cell adhesion molecules, enzymes, or structural or transport proteins associated with normal and disease-related functioning. Knowing that specific glycans can be identified using lectins, we traced the adhesion of urothelial cancerous cells to lectin-coated surfaces within a broad timescale, from the initial formation of cell attachments to the steady-state phase where cells spread on the surface. Our results showed that two lectins, namely, PHA-L and WGA, can be used to identify and differentiate specific urothelial cancer types in a label-free manner, opening the possibility of designing lectin-based biosensors that could be applied for diagnostic or prognostic purposes. Identifying specific lectins attracting cancerous cells can also be the basis for developing strategies for drug delivery that could specifically and selectively target cancer-associated glycans in tumor cells.

## Figures and Tables

**Table 1:** Specificity of lectins to glycan types identified on the cell surface.

Lectins	Detected Glycan Type	Reference
DBA	N-acetyl-α-D-galactosamine (GalNAc)	[18]
LCA	α-D-mannose (Man); α-D-glucose (Glu)	[18]
PHA-L	N-Acetyl-α-D-glucosamine (GlcNAc)	[10]
WGA	sialic acids; N-Acetyl α-D-glucosamine (GlcNAc)	[13] [10]
Con A	α-D-mannose (Man); α-D-glucose (Glu)	[49]

**Table 2:** The numbers of cells/maps/force curves recorded during SCFS measurements.

	Lectins	DBA	LCA	PHA-L	WGA	BSA
Cells						
HCV29		10/10/843	10/10/908	10/10/944	10/10/914	10/10/972
T24		10/10/892	10/10/982	10/10/958	10/10/986	10/10/986
HT1376		10/10/970	10/10/970	10/10/989	10/10/966	10/10/933

## Data Availability

The data presented in this study are available on request from the corresponding author.

## Supplementary Materials

The following supporting information can be downloaded at: https://www.mdpi.com/article/10.3390/ijms24098213/s1.
