# Supplementary material for: Bladder Cancer Cells Interaction with Lectin-Coated Surfaces under Static and Flow Conditions
Source: Int J Mol Sci. 2023 May 4;24(9):8213. doi: 10.3390/ijms24098213 (PMC10179195; doi:10.3390/ijms24098213)
Supplement: Supplementary file 1 [file ijms-24-08213-s001.zip › ijms-2346785-supplementary.pdf]

International Journal of
*Molecular Sciences*

MDPI

*Supplementary Information*

# Bladder cancer cells interaction with lectin-coated surfaces under static and flow conditions

**Renata Szydlak[1], Ingrid H. Øvreeide[2], Marcin Luty[1], Tomasz Zieliński[1], Victorien E. Prot[3], Joanna Zemła[1], Bjørn T. Stokke[2] and Małgorzata Lekka[1,*]**

[1] Department of Biophysical Microstructures, Institute of Nuclear Physics, Polish Academy of Sciences, PL-31342 Kraków, Poland; renata.szydlak@ifj.edu.pl, marcin.luty@ifj.edu.pl, Tomasz.zielinski@ifj.edu.pl, Joanna.zemla@ifj.edu.pl, malgorzata.lekka@ifj.edu.pl
[2] Biophysics and Medical Technology, Department of Physics, The Norwegian University of Science and Technology (NTNU), NO-7491 Trondheim, Norway; ingrid.h.ovreeide@ntnu.no, bjorn.stokke@ntnu.no
[3] Biomechanics, Department of Structural Engineering, The Norwegian University of Science and Technology (NTNU), NO-7491 Trondheim, Norway; victorien.prot@ntnu.no

* Correspondence: malgorzata.lekka@ifj.edu.pl; ML; bjorn.stokke@ntnu.no; BTS

**Supplementary Table S1 - Density of bladder cancer cells on lectin-coated surfaces in static conditions**

Table S1. Adhesion of bladder cancer cells to lectin-coated surfaces in static conditions expressed as a mean number of cells/ mm$^2$ ± SD.

| Coatings \ Cells | HCV29 | HT1376 | T24 |
|---|---|---|---|
| BSA | 11.06 ± 1.63 | 2.01 ± 1.84 | 4.88 ± 2.26 |
| lectins | | | |
| DBA | 64.06 ± 12.55 | 4.02 ± 1.42 | 54.30 ± 8.94 |
| LCA | 55.02 ± 14.80 | 12.07 ± 3.39 | 66.79 ± 9.08 |
| PHA-L | 86.19 ± 21.46 | 8.33 ± 2.59 | 125.26 ± 17.83 |
| WGA | 33.33 ± 4.26 | 21.55 ± 4.84 | 35.34 ± 5.81 |

**Supplementary Figure S1 - The surface density of cells attached to the uncoated glass surface**

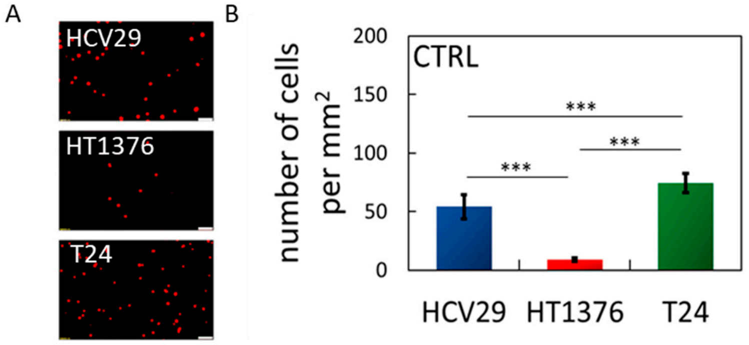


Figure S1. The surface density of cells attached to the uncoated glass surface. A) Representative fluorescence images of the bladder cancer cells labeled by Cell Tracker Red, attached to lectin-coated surfaces. Scale bar = 100 µm. B) The number of cells per mm$^2$ represented as a mean ± SD of 3 independent experiments for cells adhered to the glass surface.***$p < 0.001$.

**Supplementary Note S1 – Determination of the wall shear stress**

The bottom wall shear stresses were calculated using the microfluidic velocity profile of straight channels with rectangular cross-sections [1]. The wall shear stresses across the width of channels were calculated based on the velocity profile of Poiseuille flow in a microfluidic channel with a rectangular cross-section (height $h$ along the z-axis, width w along the y-axis):

$$v_x(y,z) = \frac{4h^2\Delta p}{\pi^3 \eta L} \sum_{n,odd}^{\infty} \frac{1}{n^3}\left[1 - \frac{\cosh(n\pi y/h)}{\cosh(n\pi w/2h)}\right]\sin\left(n\pi\frac{z}{h}\right) \tag{1}$$

where $\Delta p$ is the pressure drop over the length $L$ of the channel and $\eta$ the viscosity of the solution. The calculation of wall shear stress across the width of the channels was calculated as:

$$\tau_{xz} = \eta \left.\frac{\partial v_x(y,z)}{\partial z}\right|_{z=0} \tag{2}$$

In this calculation, the pressure drop per unit length was obtained from the relation to the volumetric flow rate $Q$:

$$Q = \frac{h^3 w \Delta p}{12\eta L}\left[1 - \sum_{n,odd}^{\infty} \frac{1}{n^5}\frac{192}{\pi^5}\frac{h}{w}\tanh\left(n\pi\frac{w}{2h}\right)\right] \tag{3}$$

An example of the variation of wall shear stress across the cross-section of a channel is shown in Supplementary Figure S2.
Plateau values of the wall shear stress, e.g. $\tau_{xy}(y = 0)$, and average values over the cross-section for the channel dimensions and various fluids are summarized in Supplementary Table S2.

**Supplementary Figure S2 - Wall shear stress across the width of a microfluidic channel**

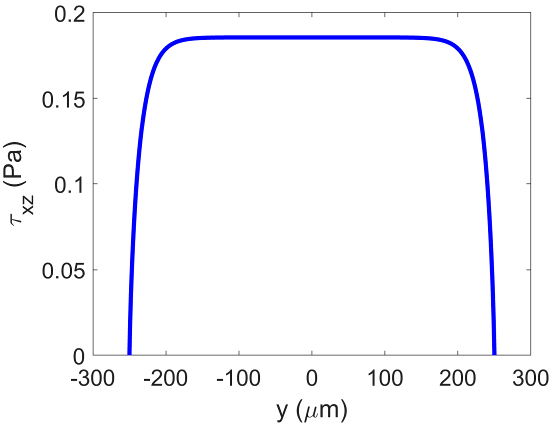


Figure S2. Wall shear stress across the width of a microfluidic channel. The wall shear stress was calculated for a fluid with a viscosity of 0.659 mPas in a channel of 500 µm width, 50 µm height, and 30 mm length at a volumetric flow rate of 3.33 µl/min.

**Supplementary Table S2 - Channel parameters and bottom wall shear stresses**

Table S2. Channel parameters and bottom wall shear stresses for culture medium RPMI 1640, DMEM, and PBS at various temperatures and channel dimensions in the employed Ibidi and custom-designed microfluidic channels.

| | | Ibidi channels | | | µ-channels |
|---|---|---|---|---|---|
| Channel parameters | Length (mm) | 50 | 50 | 50 | 30 |
| | Width (µm) | 5000 | 5000 | 5000 | 500 |
| | Height (µm) | 400 | 400 | 400 | 50 |
| Liquid at 20 °C | Type (µm) | RPMI | DMEM | PBS | PBS |
| | Temperature | 20 °C | 20 °C | 20 °C | 20 °C |
| | Viscosity (mPas) | 1.486 [a] | 1.442 [a] | 1.0219 [b] | 1.0219 [b] |
| Flow Rate | Q, (µl/min) | 300 | 300 | 300 | 3.33 |
| Wall shear stress | Plateau (Pa) | 0.0579 | 0.0562 | 0.0398 | 0.287 |
| | Average (Pa) | 0.0553 | 0.0536 | 0.0380 | 0.271 |
| Liquid at 37 °C | Type (µm) | RPMI | DMEM | PBS | PBS |
| | Temperature | 37 °C | 37 °C | 37 °C | 37 °C |
| | Viscosity (mPas) | 0.958 [c] | 0.930 [c] | 0.659 [c] | 0.659 [c] |
| Flow rate | Q, (µl/min) | 300 | 300 | 300 | 3.33 |
| Wall shear stress | Plateau (Pa) | 0.0373 | 0.0362 | 0.0257 | 0.185 |
| | Plateau (Pa) | 0.0356 | 0.0346 | 0.0245 | 0.175 |

a) The viscosities of RPMI medium with 10% added FBS and DMEM with 10% added FBS at 20 °C were estimated from the viscosity of water at 20 °C and using the same relative increase over water as reported at 37 °C [2]

b) The viscosity at 20 °C used in the calculations is 1.0219 mPa·s, as reported by Brown et at. [3]

c) The viscosities of the various media at 37 °C were adapted as reported by Poon et al. [2]

**References**:


1. Henrik Bruus, Theoretical Microfluidics, 2008, Publisher: Oxford University Press, ISBN: 978–0–19–923509–4

2. Poon, C. Measuring the Density and Viscosity of Culture Media for Optimized Computational Fluid Dynamics Analysis of in Vitro Devices. J. Mech. Behav. Biomed. Mater. 2022, 126, 105024.

3. Brown, P.H.; Balbo, A.; Zhao, H.; Ebel, C.; Schuck, P. Density Contrast Sedimentation Velocity for the Determination of Protein Partial-Specific Volumes. PLoS One 2011, 6, e26221.

**Supplementary Table S3 – Density of bladder cancer cells on lectin-coated surfaces under flow conditions**

Table S3. Adhesion of bladder cancer cells to lectin-coated surfaces in flow conditions expressed as a mean number of cells/ mm$^2$ ± SD.The adhesion under flow was conducted at a volumetric flow of 300 µl/min using RPMI medium at 37 °C in the Ibidi channels.

| Cells / Coatings | HCV29 | HT1376 | T24 |
|---|---|---|---|
| BSA | 0.13 ± 0.02 | 0.07 ± 0.01 | 0.10 ± 0.01 |
| Lectins | | | |
| DBA | 2.43 ± 0.22 | 1.00 ± 0.14 | 2.36 ± 0.33 |
| LCA | 2.17 ± 0.28 | 1.19 ± 0.30 | 2.85 ± 0.45 |
| PHA-L | 5.15 ± 0.70 | 1.66 ± 0.30 | 3.22 ± 0.28 |
| WGA | 1.66 ± 0.38 | 5.31 ± 0.34 | 3.55 ± 0.25 |

**Supplementary Figure S3 - Morphology of bladder cancer cells on lectin-coated surfaces**

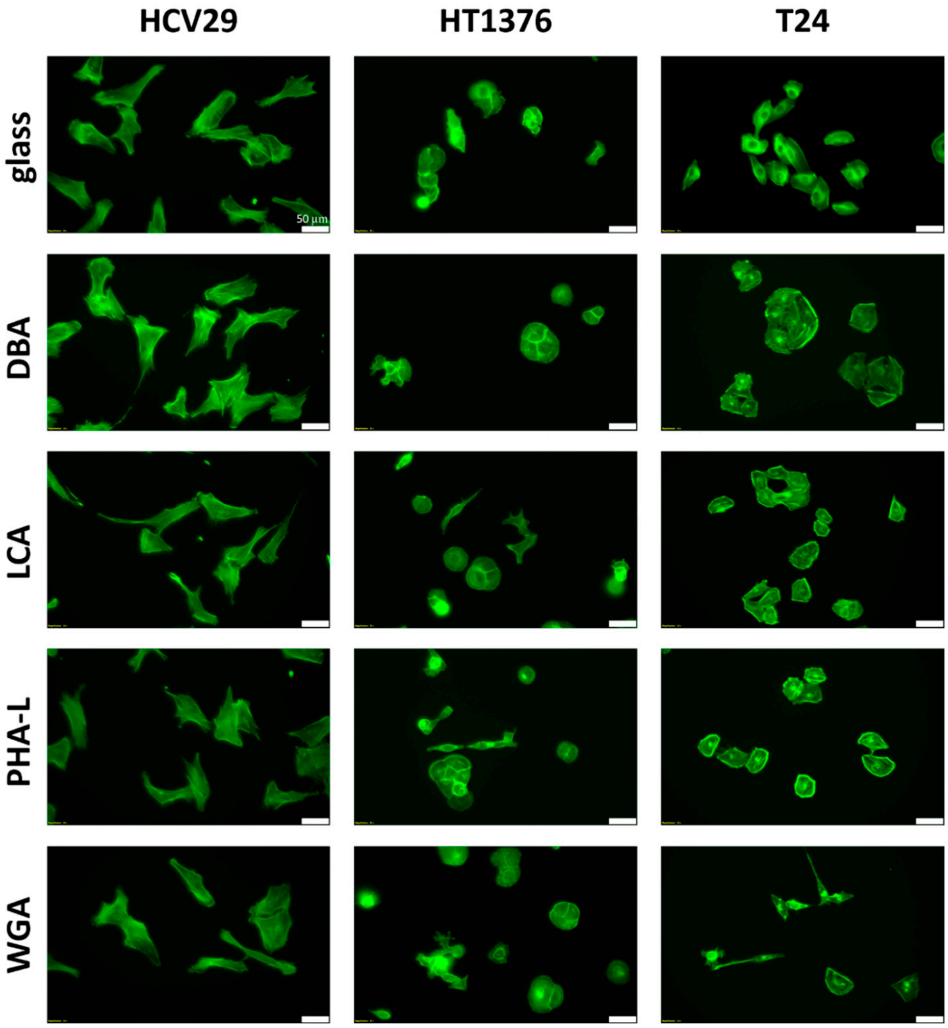


Figure S3. Morphology of bladder cancer cells cultured on bare glass and lectin-coated surfaces after 24h of culture. Fluorescent images of F-actin (labelled with phalloidin – Alexa Fluor 488 dye). Scale-bars = 50 µm.

**Supplementary Note S2 - Fluorescence scan analysis**

The collected scan of the Ibidi μ-channel surface (Suppl. Fig. S4) was analyzed with the ImageJ software (version 1.53k, https://imagej.nih.gov/ij/).

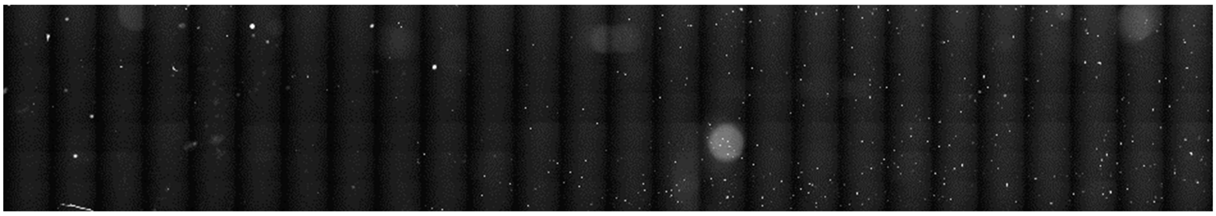

Figure S4. An exemplary image of the channel obtained using the Prior system consisting of a table enabling scanning in the x and y planes (fluorescence scan).

The "Subtract Background" operation based on the Rolling Ball algorithm removes artifacts such as background heterogeneity and some repetitive non-cell structures (Suppl. Fig. S5). The sphere radius for the rolling ball algorithm has been set to 50.0 pixels.

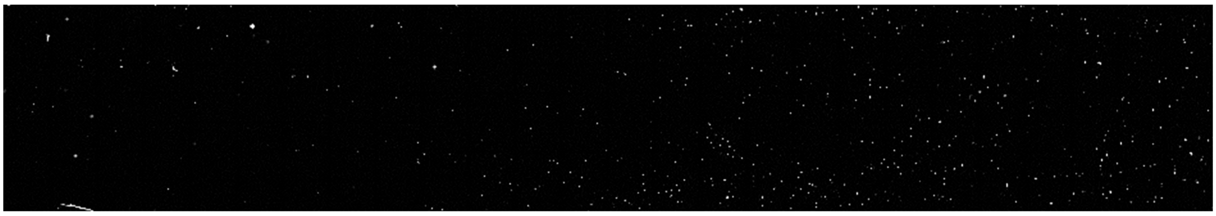

Figure S5 An exemplary image of the channel after applying the "Subtract Background" operation.

Then, binary masks covering cells located on the substrate were obtained using the Threshold method (Suppl. Fig. S6).

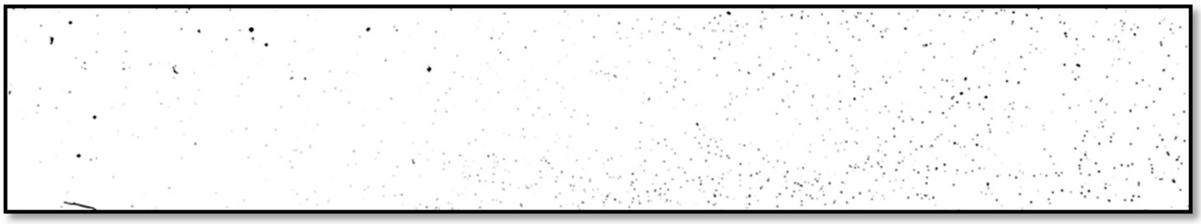

Figure S6. An exemplary binary mask obtained by the "Threshold" operation.

The number of cells that adhered to the substrate was calculated using the "Particle Analysis" option. Parameters such as "area" and "circularity" of the counted cells have been optimized and set to 500-10000 pixels and 0.20-1.00, respectively. Objects on the border of the image were excluded from the analysis.

We quantify cell adhesion to lectin-coated surfaces following the timescale of cells spreading. Single-cell force spectroscopy (SCFS) measures the adhesive properties at the initial stage of adhesion, when individual bonds are formed (time < 1 min). Considering the potential application of lectin-coated surfaces to collect cells with specific glycans on the surface, we decided to quantify the adhesion by counting the cell number after 15 minutes (during the spreading state) in static and flow conditions. The latter were conducted using a home-built system based on Ibidi-channels and custom-designed microfluidic channels.

**Supplementary Note S3. Live/dead assay**

To evaluate HCV29, T24, and HT1376 cells survival during flow experiment duration in experimental medium (mixture of RPMI 1640 and EMEM in ratio 2:1), 04511 Cell stain double staining kit (Sigma Aldrich) was applied. Before use, 10 µl Solution A and 5 µl Solution B were dissolved in 5 mL PBS to prepare the staining solution. All cell lines were plated 24 hours before the experiment on 12 well plates at a density of 100.000 per well, suspended in 1 mL of medium. After 24 hours from plating, the medium was changed to a fresh culture medium and experimental medium to investigate the effect of the flow medium on cell survival. After 1 hour of conditioning, wells with cell cultures were washed with PBS to remove residual esterase activity. Then samples were rinsed with 1 mL staining solution per well and incubated at 37 ºC for 15 min. Cell imaging was performed using a fluorescence microscope with excitation at 490 nm for viable cells and 545 nm for dead cells were observed. Double Staining Kit differentiates viable (calcein-AM) and dead propidium iodide (PI) cells. Calcein-AM, an acetoxymethyl ester of calcein, is cell membrane permeable and exhibits lipophilic characteristics but does not emit fluorescence. Inside the living cell, the calcein is obtained from Calcein-AM by esterase and shows strong green fluorescence emission (excitation: 490 nm, emission: 515 nm).
On the other hand, intercalation of PI with DNA double helix is possible only in case of loss of permeability of cell membrane, which is correlated with cell death cascade. Staining emits red fluorescence (excitation: 535 nm, emission: 617 nm). Calcein and PI-DNA can be excited with 490 nm, simultaneously showing viable and dead cells. 545 nm excitation reveals only dead cells. The recorded fluorescent images of stained cells are shown in Supplementary Figure S7).

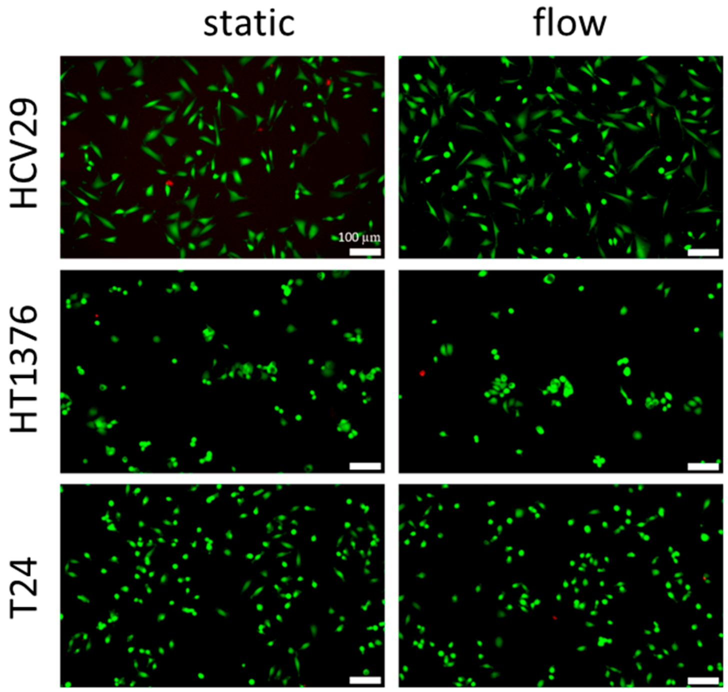

Figure S7. Live/dead analysis of bladder cancer cells using double staining of Calcein AM (green) and Propidium Iodide (PI) (red) after 1h growth in flow medium compared to the control group (i.e., cells were cultures in the corresponding medium in static conditions). Scalebars = 100 μm.

**Disclaimer/Publisher's Note:** The statements, opinions and data contained in all publications are solely those of the individual author(s) and contributor(s) and not of MDPI and/or the editor(s). MDPI and/or the editor(s) disclaim responsibility for any injury to people or property resulting from any ideas, methods, instructions or products referred to in the content.
